# Effect of electrical energy on the efficacy of biofilm treatment using the bioelectric effect

**DOI:** 10.1038/npjbiofilms.2015.16

**Published:** 2015-09-23

**Authors:** Young Wook Kim, Sowmya Subramanian, Konstantinos Gerasopoulos, Hadar Ben-Yoav, Hsuan-Chen Wu, David Quan, Karen Carter, Mariana T Meyer, William E Bentley, Reza Ghodssi

**Affiliations:** 1 MEMS Sensors and Actuators Laboratory, Institute for Systems Research, University of Maryland, College Park, MD, USA; 2 Department of Electrical and Computer Engineering, University of Maryland, College Park, MD, USA; 3 Fischell Department of Bioengineering, University of Maryland, College Park, MD, USA; 4 Department of Chemical and Biomolecular Engineering, University of Maryland, College Park, MD, USA

## Abstract

**Background/Objectives:**

The use of electric fields in combination with small doses of antibiotics for enhanced treatment of biofilms is termed the ‘bioelectric effect’ (BE). Different mechanisms of action for the AC and DC fields have been reported in the literature over the last two decades. In this work, we conduct the first study on the correlation between the electrical energy and the treatment efficacy of the bioelectric effect on *Escherichia coli* K-12 W3110 biofilms.

**Methods:**

A thorough study was performed through the application of alternating (AC), direct (DC) and superimposed (SP) potentials of different amplitudes on mature *E. coli* biofilms. The electric fields were applied in combination with the antibiotic gentamicin (10 μg/ml) over a course of 24 h, after the biofilms had matured for 24 h. The biofilms were analysed using the crystal violet assay, the colony-forming unit method and fluorescence microscopy.

**Results:**

Results show that there is no statistical difference in treatment efficacy between the DC-, AC- and SP-based BE treatment of equivalent energies (analysis of variance (ANOVA) *P*>0.05) for voltages <1 V. We also demonstrate that the efficacy of the BE treatment as measured by the crystal violet staining method and colony-forming unit assay is proportional to the electrical energy applied (ANOVA *P*<0.05). We further verify that the treatment efficacy varies linearly with the energy of the BE treatment (*r^2^* =0.984). Our results thus suggest that the energy of the electrical signal is the primary factor in determining the efficacy of the BE treatment, at potentials less than the media electrolysis voltage.

**Conclusions:**

Our results demonstrate that the energy of the electrical signal, and not the type of electrical signal (AC or DC or SP), is the key to determine the efficacy of the BE treatment. We anticipate that this observation will pave the way for further understanding of the mechanism of action of the BE treatment method and may open new doors to the use of electric fields in the treatment of bacterial biofilms.

## Introduction

Bacterial biofilms are complex communities comprising of bacteria and extracellular matrix.^1–3^ The composition and organisation of biofilms limits diffusion of molecules, including antibiotics, through the structure and into the biofilm or out to the bulk fluid.^4^ Bacteria in biofilms are also more likely to exchange their genes resulting in much higher antibiotic resistivity than bacteria in suspensions.^3^ Because of these factors, the treatment of biofilms is particularly challenging, often requiring antibiotic doses in excess of 500 times than those needed for the same bacteria in planktonic states.^5–9^ However, such high doses are impractical because they dramatically increase the risk of harmful side effects and contribute to the proliferation of multidrug-resistant strains.^10–12^ A great need exists to enhance the efficacy of existing antibiotics and develop innovative tools to limit dose.

A promising method to increase the efficacy of antibiotics on biofilms is a combinatorial treatment based on applying electrical signals in combination with low doses of antibiotic, also termed the ‘bioelectric effect’ (BE).^13–15^ Costerton *et al*.^9^ demonstrated improved biofilm treatment through the application of either direct or alternating current (DC or AC) electric fields.^16–24^ Details of the fundamental mechanisms of the bioelectric effect are still under investigation, and divergent hypotheses have emerged based on the type of the applied field. In the case of a DC voltage, the generation of radicals owing to media electrolysis is suggested as a principal factor.^17,18,25^ In addition, some reports describe enhanced efficacy owing to improved antibiotic binding to biofilms^14,26^ and enhanced biofilm detachment^27^ from an external DC electrostatic force. In the case of the AC treatment, results indicate increased permeability of the exopolysaccharide matrix because of locally charged molecular vibrations.^24^ Other reports note augmented effects from thermal stimuli^19^ as well as electrolysis of the medium.^26^ In general, investigating the mechanisms that underlie the bioelectric effect on biofilms is difficult owing to their complex structures and the diverse stimuli.^26^

Given the divergent nature of the reports on the attributable mechanisms of action for DC and AC fields independently, a hypothesis that was examined in this work is whether their superposition could result in a synergistic treatment effect, by combining the reported benefits of both DC and AC fields, namely increased permeability of the exopolysaccharide matrix, and media electrolysis, biofilm detachment and improved antibiotic binding, respectively. To test this hypothesis, we treated biofilms with antibiotics under the application of a superpositioned (SP) field containing both AC and DC components. Interestingly, we observed that the treatment efficacy of the SP-BE was the linear sum of the individual treatment efficacies of the AC-BE and DC-BE. As the total energy of the SP-BE was the linear sum of the AC-BE and DC-BE, we investigated the effect of total electrical energy on BE treatment efficacy and established that the energy provided to the BE was the governing factor that dictated the efficacy of the treatment. Despite the number of studies of both AC and DC fields and their apparent successes, to date, no studies on the effect of the total electrical energy have been conducted.

We treated *Escherichia coli* biofilms^28^ with DC, AC and SP electric fields in combination with the antibiotic gentamicin.^29^ We tested the effect of the electrical signal energy on the efficacy of the bioelectric effect by applying either a DC field, an AC field or an SP field, in combination with the antibiotic gentamicin to 24-h mature biofilms. Three sets of experiments were performed and the efficacy of treatment was measured: (i) the amplitudes of the three different electrical signals (AC, DC and SP) were chosen such that the magnitude of energy of the SP signal was the sum of the magnitudes of the DC and AC signal energies, (ii) the amplitudes of the AC, DC and SP potentials were chosen such that each signal had the same magnitude of energy when applied over a period of 24 h and (iii) increasing energies of the AC electrical signal was applied over a period of 24 h. The effectiveness of the BE treatment was quantified by the crystal violet (CV) staining method and the live bacterial density results as measured by the colony-forming unit (CFU) assay. As the voltages selected were less than or close to 0.82 V, we were able to avoid electrolysis of the surrounding medium. The concentration of gentamicin (10 μg/ml) used in our experiments is significantly lower than what is typically necessary for biofilm treatment (500–5000 times the concentration compared with the minimum inhibitory concentration (MIC) of suspended bacteria, MIC =2–5 μg/ml).^9^ Our experiments resulted in two key conclusions. First, we observed that biofilm BE treatment with an SP signal of higher electrical energy that was the sum of two smaller AC and DC energies resulted in a net treatment efficacy that was equivalent to the sum of the individual AC and DC treatment efficacies. Second, the application of electrical signals (DC or AC or SP) of the same energy in combination with a fixed concentration of gentamicin resulted in equivalent treatment efficacies. These results reveal that the signal energy, and not the type of electrical signal (AC or DC or SP), is the primary parameter that governs the mechanism of action of the BE. These conclusions were further confirmed when varying the BE energy, by changing the amplitude of an AC potential, resulted in a linear change in efficacy.

The results presented in this work bring to light that the mechanism of action of the BE is not different for AC or DC or SP fields for potentials <1 V, as reported previously. We hypothesise that the electrical energy applied to the treatment in the form of the DC, AC or SP signals provides the charged antibiotic molecule with additional drift that results in the enhanced efficacy of this treatment. The linear dependence of the BE on the electrical energy, enables deterministic modification to the treatment. In addition, BE dependence on the energy and not the signal type allows for more efficient utilisation of nearby electronic resources. For example, in an *in vivo* BE treatment system, generation of on-chip AC signals from nearby electronics can be achieved more easily with higher efficiency as compared with generation of a pure DC potential. It also opens up the opportunity to transmit wireless power in the form of an AC signal so that future designs of *in vivo* sensor-treatment platforms can include electronics for inductive power transmission. True understanding of the mechanism of action of the BE will thus allow for more flexibility and ease of integration of the BE into various applications in both the clinical and environmental fields.

## Materials and Methods

### Cuvette test apparatus

**Figure 1:**
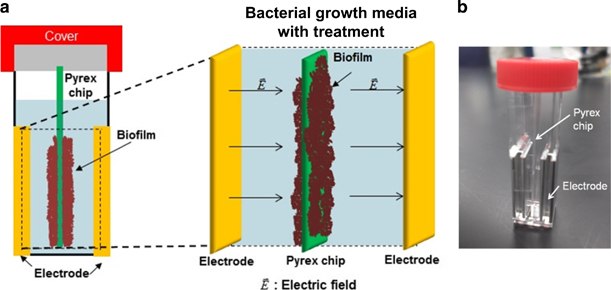
(**a**) Schematic of experimental setup. Glass chips/coupons placed inside electroporation cuvettes were used as the growth surface for bacterial biofilms. The electrodes of the cuvette were used for easy application of electric fields. (**b**) Photograph of electroporation cuvette with glass coupon placed inside it.

The experimental apparatus for BE studies was designed to ensure uniform electric fields, while retaining access for sensing and fluid manipulation. Cuvettes (P460-50, Invitrogen Inc., Carlsbad, CA, USA) with parallel stainless steel electrodes forming two of the walls were used to apply a near-uniform electric field inside the cuvette (Figure 1). The gap between the two electrodes was 0.4 cm. A 500-μm thick Pyrex wafer was diced into chips (or coupons) with dimensions of 0.8 cm×4 cm (width×length). The diced glass chip was inserted upright into the cuvette between the two electrodes as shown in Figure 1b. The chip served a consistent area for biofilm growth. The electrical signal was provided by a function generator (33220A, Agilent Inc., Santa Clara, CA, USA) with a coaxial cable connection to the electrical contact board.

### Biofilm growth

A bacterial suspension was prepared from *E. coli* K-12 W3110 [F^−^ λ^−^ in(*rrnD*-*rrnE*)] samples^28^ stored in a freezer maintained at −80 °C and inoculating in 5 ml of fresh lysogeny broth (LB). The suspension was cultured at 37 °C in a 250 r.p.m. shaker for 18 h. The culture was re-inoculated into fresh LB to achieve optical densities (OD_600_) in the range of 0.20–0.25. Then 1 ml of this culture was placed in each cuvette with a glass chip for 24 h of biofilm growth. *E. coli* biofilms were formed on the Pyrex chips for 24 h in LB medium at room temperature.^17,18,30^ The glass chips with pre-formed 24-h *E. coli* K-12 W3110 biofilms were transferred to a new set of cuvettes containing 1 ml of 10 μg/ml of gentamicin in LB. Electric fields of varying strengths, discussed in the section below, were applied for 24 h to the biofilm-containing cuvettes. The biofilms were investigated by performing CFU assays for viable cell density, optical microscopy and crystal violet staining for total biomass quantification.

### Crystal violet staining (total biomass quantification)

After applying treatment to the cuvettes for 24 h, the glass chips were removed from the cuvette and rinsed with deionized (DI) water to remove non-adherent bacteria. Quantification of remaining biofilm on the chips was achieved by staining each chip for 15 min with 0.1% crystal violet,^31^ after which each chip was gently immersed and rinsed sequentially in four beakers of DI water to remove unbound crystal violet. Following this, the stained biofilms were resuspended in a 1 ml solution of 80% ethanol and 20% acetone for 30 min.^31^ The optical density (OD_540_) of this solution was measured with a spectrophotometer (Molecular Devices, Sunnyvale, CA, USA, LLC). The final OD_540_ of the crystal violet released from biofilms is proportional to the total biomass growth on the chip.^31^ Each experiment was repeated multiple times and the results were averaged.

### CFU assay (Viable cell quantification)

Following treatments, the chips were vortexed for 2–3 min in 1 ml fresh LB to re-suspend biofilms.^32^ On the basis of the OD_600_ of the solution, dilution ratios were selected for the CFU assay,^31^ and 20 μl of resuspended biofilm solution was plated on sterilised LB agar gel plates that were prepared with 25 g/l of LB and 15 g/l of agar. The plates were placed in an incubator overnight at 37 °C. The density of live bacteria (CFU/ml) on each plate was calculated on the basis of the number of CFU, the dilution ratio and the original volume (20 μl) of the biofilm solution. Each experiment was repeated multiple times and the results were averaged. The error in the results represents the standard deviation from the average live bacterial density.

### Fluorescence microscopy imaging

After biofilm formation or treatment, biofilms were rinsed with DI water and stained with the Filmtracer LIVE/DEAD Biofilm Viability Kit (Molecular Probes, Inc., Eugene, OR, USA), using equal proportions of SYTO9 and propidium iodide diluted in 5 ml of DI water. After 15 min, the biofilms were rinsed again with DI water and imaged using fluorescence microscopy (Olympus BX60, Center Valley, PA, USA). One spot was imaged for each glass coupon; images were obtained close to the centre point of each glass chip where the biofilm was observed to be the thickest. As the viability stain assay was not calibrated for biofilms formed using this experimental setup, quantitative viability data are not presented here.

### Intensity of the electric field used for treatment with different energies

By investigating biofilm growth variation while applying only an electric field to *E. coli* K-12 W3110 suspensions, the largest electric field intensity that does not cause electrolysis was determined. A total 1 ml of *E. coli* suspension (OD_600_ 0.20–0.25) in LB was placed in the cuvettes. The two DC electric field intensities investigated were 2 and 1.25 V/cm (corresponding to 0.8 and 0.5 V applied over the 0.4 cm distance between the electrodes, respectively). These values were selected on the basis of the potential limit of electrolysis, which occurs between 0.82 and 1 V.^33,34^

As the 2 V/cm DC electric field (corresponding to 0.8 V for the cuvette setup) often induced significant visually observable electrolysis of the media, electric field intensities close to 1.25 V/cm (corresponding to 0.5 V for the cuvette setup) was chosen as the intensity of the DC electric field to be used in this work. The AC field component was also chosen to be 1.25 V/cm to prevent electrolysis of the media. The frequency of the AC electric field (10 MHz) was chosen on the basis of previous work in literature.^19,24^ However, it was observed that slightly larger AC potentials, up to 0.9 V at 10 MHz, did not result in any significant electrolysis. We hypothesise that this is owing to the rapidly changing electric field wherein the peak AC voltage is attained only for a very small duration of time, which is not sufficient to cause bulk hydrolysis. The SP electric field used in this work was the superposition of the 0.5 V DC field with the 0.5 V AC field at 10 MHz.

### Electrical amplitude calculations for treatment with equivalent energies

To compare the effect of different types of electrical energy signals on biofilm treatment using the bioelectric effect, DC, AC and superimposed DC and AC fields of equivalent energies were applied in combination with 10 μg/ml of gentamicin to 24-h biofilms. The average energy *E* of the signals were calculated using the equation below^35^

(1)Esignal=1R∫0T(A+Bsin(ωt))2dt

where *A* is the amplitude of the DC potential, *B* is the amplitude of the AC component of the signal, *T* is the duration of application of the electrical signal, *R* is the resistance of the system and *ω* is the frequency of the AC signal.

Using equation (1), the energy of the SP-BE treatment (0.5 V DC and 0.5 V AC at 10 MHz) can be calculated as shown in equation (2) below. The resistance of the system is assumed to be primarily from the glass coupon. Using the resistivity of Pyrex glass as 4 MΩ-m, the resistance of a glass coupon is calculated to be 4,000 GΩ.

(2)ESP−BE=1R∫0T(0.5+0.5sin(ωt))2dt

The amplitudes of the pure DC signal and pure AC signal at 10 MHz can then be calculated such that equation (3) below was satisfied. We assume that *R* is constant across all the experiments.

(3)ESP−BE=EDC−BE=EAC−BE

The amplitudes of the DC and AC signals were calculated to be 0.613 and 0.866 V, respectively. The DC amplitude was well below the threshold of electrolysis of 0.82 V; however, the amplitude of the AC field was calculated to be slightly higher than that of 0.82 V. Nevertheless, application of an AC field of 0.866 V at 10 MHz did not result in significant electrolysis.

### Electrical amplitude calculations for treatment with varying energies

To demonstrate the relationship between applied BE energy and the efficacy of treatment, increasing energies of the same type of electrical signal was applied in combination with 10 μg/ml of gentamicin to 24-h mature biofilms. Varying amplitudes of a 10 MHz AC signal were used and the energy of the applied potential was calculated using equation (1). The potentials chosen were in the range of 0–0.9 V, within the limit of electrolysis, to avoid bulk electrolysis of the media. Specifically, four amplitudes of the AC signal: 0 V (control), 0.3, 0.6 and 0.866 V were applied to 24-h mature *E. coli* biofilms in combination with 10 μg/ml of the antibiotic for 24 h. The total biomass quantified after treatment with the varying electrical energies is normalised to the control to successfully and reliably combine multiple runs of the experiment. A linear fit of the data was performed using Origin Pro software (OriginLab Corporation, Northampton, MA, USA).

The data were inspected by an outlier checker programme (GraphPad Software, La Jolla, CA, USA, *α*=0.05) to eliminate outliers from the raw data. With the data, we performed analysis of variance (ANOVA) to investigate the significance of each experimental result. This method was applied to all the experiments.

## Results

### Effect of varying energies and field types

#### Results for CFU assay

**Figure 2:**
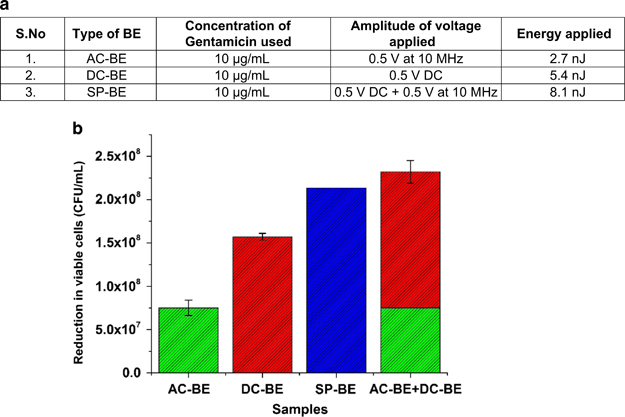
(**a**) Table summarizing the voltages and energies used to test the effect of various energies of different signals on BE treatment efficacy to treat mature *E. coli* biofilms. (**b**) Plot showing the reduction in viable cells as measured using the colony-forming unit (CFU) assay method. Treatment with SP-BE, that has twice the energy as the AC-BE or DC-BE, results in almost twice the reduction in viable cells (*N*=4 for each experiment). Furthermore, the reduction in the SP-BE viable cell count is not significantly different from the linear sum of the reduction in the AC-BE and DC-BE viable cell count. The error bars represent the standard deviation across the repeats of the experiments. The error bar for the SP-BE is not large enough to be visible at this scale. AC, alternating current; BE, bioelectric effect; DC, direct current; SP, superpositioned field.

To test the efficacy of the BE treatment, 24-hour mature *E. coli* K-12 W3110 biofilms were subjected to different fields. The concentration of the antibiotic was maintained at 10 μg/ml across all the treatments. The amplitudes of the different electrical signals—namely AC, DC and SP, used for the treatment are listed in Figure 2a. The viable cell counts of the *E. coli* K-12 W3110 biofilms exposed to the BE treatments of different energies were analysed using the CFU assay method. The reduction in viable cells (*R*) for each of the BE treatments was then calculated by subtracting the viable cell count of the untreated control biofilms. A plot of the reduction in viable cells is plotted for the different BE samples in Figure 2b.

It is interesting to note that the reductions in biomass as measured using the CFU count method are proportional to the net energy applied to the BE treatment. As shown in the figure, the reduction in viable cells due to the AC-BE is the lowest (*R*_AC-BE_=7.5×10^7^ CFU/ml) as it provides the lowest electrical energy during treatment. This is followed by the DC-BE, which shows a reduction of ~1.6×10^8^ CFU/ml (*R*_DC-BE_). Although the DC-BE has the same signal amplitude as the AC-BE (0.5 V), it provides twice the energy to the BE treatment, which results in twice the reduction in viable cells (Figure 2b). The SP-BE, which is the superposition of the AC-BE and the DC-BE signals, results in the highest reduction in viable cells of 2.2×10^8^ CFU/ml (*R*_SP-BE_). Moreover, treatment with the SP-BE energy (*E*_SP-BE_=*E*_AC-BE_+*E*_DC-BE_) results in a reduction in viable cells (*R*_SP-BE_) that is not significantly different from the linear sum of the reduction in viable cells owing to the AC-BE (*R*_AC-BE_) and DC-BE (*R*_DC-BE_) or *R*_SP-BE_=*R*_AC-BE_+*R*_DC-BE_ as plotted in Figure 2b below. This experiment verified that the superposition of the AC and DC signals did not result in a synergistic treatment effect as hypothesised. However, the results suggest that the BE could depend on the energy provided to the treatment. Hence, further experiments to understand the effect of varying energy on the efficacy of the BE treatment were performed.

#### Results for CV staining method

**Figure 3:**
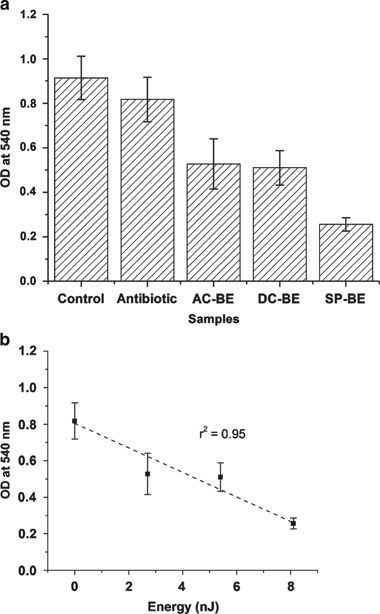
(**a**) Results of total biomass quantification using the crystal violet staining method. (**b**) Linear fit of the total biomass for the different energies provided during BE treatment. Plots show the OD at 540 nm after staining the treated biofilms with CV. Results show that the SP-BE shows a 71% reduction in bacterial biomass as compared with the untreated control (analysis of variance *P*<0.05). The SP-BE, which has higher energy as compared with the AC-BE or DC-BE is also more effective in treating biofilms. The data presented are the average OD_540_ and the error bars represent the standard deviation over repeated experiments (*N*=6 in each experiment). AC, alternating current; BE, bioelectric effect; DC, direct current; OD, optical density; SP, superpositioned field.

After treatment with the different BEs for 24 h, the biofilms were quantified using the CV staining method. Negative controls, i.e., treatment with only antibiotic (no electric field) and pure LB (no antibiotic or electric field) were also performed. The results of this experiment are shown in Figure 3.

Figure 3a plots the total biomass of the biofilms for the various treatments applied. Treatment with only antibiotics at near MIC concentrations resulted in a very small, if negligible, reduction in biomass. This is expected as biofilms are known to have increased antibiotic resistance and require at least 500–5,000 times the MIC dosage of antibiotics for effective treatment.^1,36–38^ Treatment with the AC-BE, DC-BE and the SP-BE resulted in significant reduction in bacterial biomass as compared with the controls (ANOVA *P*<0.05). Approximately 50% reduction in total biomass is observed when biofilms are treated with the DC-BE, as compared with the untreated controls (ANOVA *P*<0.05). Treatment with the SP-BE that has almost 1.5 times the energy as the DC-BE resulted in a significant decrease in total biomass (ANOVA *P*<0.05) of 50% over the DC-BE, or an overall decrease of almost 71% as compared with the untreated control.

The total biomass, as measured using the CV staining method, is also plotted as a function of the total energy applied with the BE (Figure 3b). The biofilms treated with only 10 μg/ml of the antibiotic gentamicin is plotted as the control (treatment with no electrical energy). Again a strong linear dependence of the treatment efficacy on the applied BE energy is observed (*r*^2^=0.950). These results further demonstrate that the superposition of the two types of fields, AC and DC, during the BE treatment does not result in a synergistic effect. Rather, the energy of the electrical signal may have a key role in determining the efficacy of the treatment.

### Effect of varying field types at equal energies

#### Results for CV staining method

**Figure 4:**
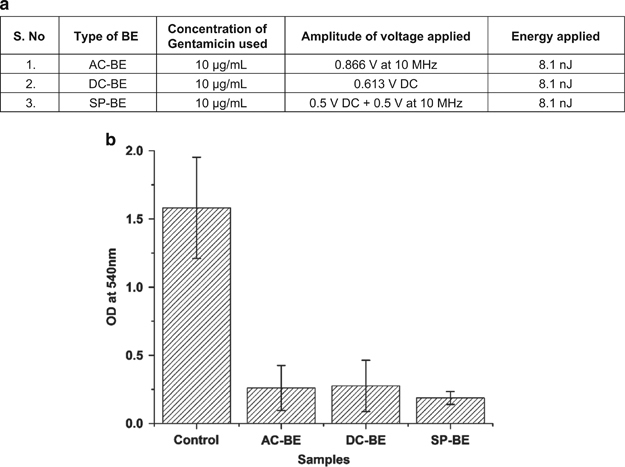
(**a**) Table summarizing the magnitude of voltages used to test the effect of equivalent energies of different signals on BE treatment efficacy to treat mature *E. coli* biofilms. (**b**) Figure plotting OD measured at 540 nm for various biofilms samples treated with BE of equivalent energies. The energy of the electrical signal dictates the efficacy of the BE as observed by the similar reduction in total biomass for the AC-BE, DC-BE and SP-BE treatments. The error bars represent the standard deviation of the experiments performed across eight samples (*N*=8 for each experiment). AC, alternating current; BE, bioelectric effect; DC, direct current; OD, optical density; SP, superpositioned field.

To verify whether the electrical energy supplied to the BE treatments is the dominant factor affecting the efficacy of the BE treatment, AC-BE, DC-BE and SP-BE treatments of the same energy were applied to 24-h mature *E. coli* biofilms over 24 h. The energies and potentials of the AC, DC and SP signals were established using equation (3), and are tabulated in Figure 4a. After treatment, the biofilms were stained using the CV staining method and the OD at 540 nm was recorded. A plot of the OD_540_ for the different BE treatments and the control is shown in Figure 4b.

As seen from Figure 4b, the AC-BE, DC-BE and SP-BE treatments with equivalent energies result in a similar reduction in bacterial biomass (ANOVA *P*>0.05). On average, the AC-BE resulted in an 83.57% decrease, the DC-BE treatment resulted in an 82.55% decrease and the SP-BE resulted in an 88.15% decrease in total biomass as compared with the untreated control. Overall, the BE treatments with equivalent energies resulted in an 84.76±5.32% average reduction of total biomass as compared with the control. This illustrates that the type of electrical signal used (AC, DC or SP) does not affect the efficacy of the BE treatment. Furthermore, these results illustrate that the BE energy applied is the prime factor in determining the treatment efficacy.

#### Results for fluorescence microscopy

**Figure 5:**
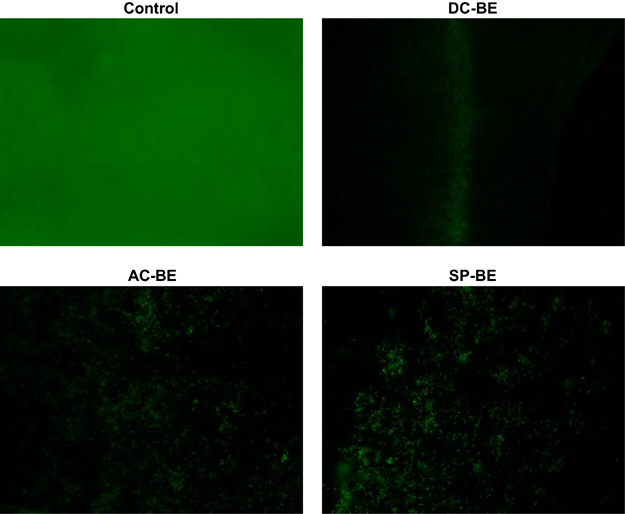
Fluorescence microscopy images of the biofilm grown on the glass coupon after treatment with DC-BE, AC-BE and SP-BE as compared with untreated biofilms (control). The BE-treated biofilms result in a similar reduction in biomass as observed from the images. AC, alternating current; BE, bioelectric effect; DC, direct current; SP, superpositioned field.

To visualise the effect of the BE treatments on the biofilms, the treated biofilms were stained using Filmtracer LIVE/DEAD Biofilm Viability Kit and imaged under the fluorescence microscope. The images for the control, DC-BE, AC-BE and SP-BE biofilms are shown in Figure 5. The control biofilm is the most dense, whereas the BE-treated samples result in almost complete removal of biofilm. Furthermore, the three BE-treated biofilms result in a similar reduction of biomass as observed from the images of Figure 5.

### Effect of varying energies of equal field types

**Figure 6:**
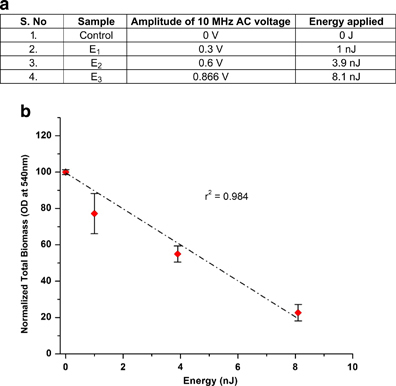
(**a**) Table summarizing the magnitude of AC voltage used to test the effect of increasing energies of the same type of signal on BE treatment efficacy to treat mature *E. coli* biofilms. (**b**) Plot showing the linear relationship between the total biomass of the biofilms as measured using CV staining method and the voltage or energy of the electrical signal applied. The error bars represent the standard deviation across the repeats (*N*=5 repeats) for each experiment. AC, alternating current; BE, bioelectric effect; CV, crystal violet.

To determine the relationship between the BE energy and the treatment efficacy of the BE, mature *E. coli* biofilms were treated to different electrical energies of a similar signal type. As observed from the results presented in Figure 4b, biofilm reduction does not depend on the type of electrical signal used for the BE treatment, i.e., AC or DC or SP. Hence, for these experiments, we arbitrarily chose to use AC fields. Specifically, varying amplitudes of a 10 MHz AC signal was used in combination with 10 μg/ml of the antibiotic gentamicin. The magnitudes of the voltages used are tabulated in Figure 6a. The biofilms were then stained using the CV staining method and the OD_540_ was measured (*N*=5 repeats per data point).

The results of increasing energy on BE treatment efficacy as measured using the CV staining method is plotted in Figure 6b. We observe a decrease in total biomass with increasing energy supplied to the BE treatment. Specifically, the application of energy *E*_3_=8.1 nJ, through the application of a 10 MHz AC voltage of 0.866 V, results in an 80 % reduction in bacterial biomass. This correlates very well with the results previously presented in Figure 4, wherein application of the same energy level *E*_3_ through the use of either an AC, DC or SP voltage results in a similar decrease in total biomass. These results when taken together validate our hypothesis that the energy of the electrical signal is the primary factor in determining the efficacy of the BE treatment.

## Discussion

In this work, the efficacy of treating biofilms with an electric field applied concurrently with antibiotic treatment was evaluated as a function of energy and type of electric signal (AC, DC or SP). We hypothesised that the superposition of DC and AC fields would enable the simultaneous application of all mechanisms previously attributed to both DC and AC currents individually. That is, a DC electric field can create a non-uniform distribution of electrolytes^26,39^ and an AC field can increase biofilm permeability.^24^ However, the results obtained in this work support the conclusion that the biocidal effects of the antibiotic can be improved to a similar extent when different types of electrical fields of equivalent energies are applied. Overall, our data demonstrate that the enhancement of biofilm treatment when antibiotics are combined with electric fields is primarily because of the additional energy provided to the treatment and not owing to the type of electric signal used. Furthermore, we believe that the additional energy provided to the treatment allows for a stronger directed flow of the charged antibiotic molecule into the biofilms that results in the enhanced treatment efficacy. Hence, when higher energy is provided to the treatment either in the form of increased electrical potential or in the form of longer durations of treatment we expect to observe larger reduction in biomass.

The magnitude of the voltages applied in this work is lower than the threshold potential of biological electrolysis of the medium (0.82 V).^33^ This was done to avoid the generation of hazardous radicals.^17^ Often, in previous work, the applied voltages for analysing the bioelectric effect have been above 0.82 V (typically in the range of 2–5 V/cm).^9,25,26^ In the study performed by Costerton *et al*.,^9^ 5.0 times the MIC of tobramycin was used in combination with an electric field of 5 V/cm for 48 h to treat *Pseudomonas aeruginosa* biofilms. Treatment with this high-energy combination therapy resulted in almost a complete kill of the viable cells (<10^2^ viable cells per cm^2^; ~10^6^ reduction in viable cells per cm^2^). In another detailed study,^22^ the electrical enhancement of different classes of antimicrobials (antimicrobial concentration range of 1.0–32.0 times the MIC) on various bacterial biofilms was studied. Treatment with 200 and 2,000 μA in combination with the antimicrobials resulted in ~10^2^–10^4.5^ CFU/cm^2^ mean reduction in viable cells. Correspondingly, media electrolysis owing to the high external electric fields used has often been cited as one of the major contributors to the enhanced biocidal effects observed under applied DC currents.^17,18,25^ Such electrolysis leads to oxidant generation and media decomposition. These factors make the direct attribution of specific mechanisms of action difficult.^13,26^ Furthermore, they make integration of such high voltage treatments in *in vivo* systems impossible. In comparison, in our work, we applied only 2.0 times the MIC of gentamicin in combination with low SP fields over 24 h and observed a decrease of 2×10^8^ CFU/ml.

In this work, we present effective treatment using voltages below those needed for electrolysis. We tested for bulk pH changes owing to electrolysis, using a pH indicator (#36828, Fluka Analytical) which changes colour in the pH range of 4–10, after application of the SP-BE field to unbuffered LB media for 24 h; results reveal no statistical difference relative to the control (no applied potential). This is in contrast to significant bulk pH change measured when the electrolysis threshold voltage of 0.82 V DC was applied (Supplementary Figure S1, ANOVA *P*<0.05). Samples subjected to AC or DC fields used here also exhibited no visible signs of bulk electrolysis relative to the control. We thus conclude that the application of electric fields used in this work does not induce any significant fluidic electrolysis.

We also note that thermal effects can be induced by applying external fields, which might lead to misinterpretation of data. Temperatures greater than 45 °C result in biocidal effects as enzymes and proteins essential to biofilm growth processes are denatured.^40^ Several studies, however, have reported that local heating from applied AC signals with field intensities of 2 V/cm was less than 1 °C for a 24-h treatment. This temperature change did not affect bacterial growth.^19,24^ As the applied field intensity in this work was lower than previous reports (2 V/cm), we did not pursue these effects for detailed study.

Finally, we note that the bioelectric effect is applicable to a broad range of microorganisms; hence, it cannot be generalised. Previous studies demonstrate that the bioelectric effect can be extended to different species of bacteria and various antibiotics.^22,41,42^ The enhancement in efficacy of the antibiotics through the use of electrical potentials is observed to be different for different bacterial species and is known to depend on multiple parameters like the type of antibiotic, the antibiotic concentration and the electrical energy applied.^22,23,43^ However, we suggest that when all other experimental parameters (bacterial species, antibiotic and antibiotic concentration) are kept constant, for the same magnitude of electrical energy applied, irrespective of the type of electrical signal, a similar increase in efficacy of treatment is expected to be observed.

In summary, significantly improved treatment of biofilms was demonstrated by using electric fields in conjunction with the antibiotic gentamicin. It was observed that the BE supplied with higher electrical energy induced greater biofilm reduction than the BE with lower electrical energy for applied voltages less than the media electrolysis voltage. We further note that the type of electrical signal did not appear to affect the efficacy of the treatment indicating that the mechanism of action is not different for DC versus AC signals in this range of potentials (applied voltages less than 1 V). We suggest that the enhanced treatment efficacy of any BE treatment (AC, DC or SP) is primarily owing to the energy provided to the treatment that allows for either increased permeability of the membranes or the apparent improved diffusion of the charged antibiotics or both. We highlight that the intensity of the fields utilised here was below the electrolysis potential of the biological fluid. Hence, applications of this technique would minimise generating harmful radicals due to media electrolysis, enabling future integration in *in vivo* systems.

## Conclusions

The results presented here show that the energy of the BE signal dictates the efficacy of the BE treatment for a fixed antibiotic concentration. Treatment with electrical signals whose energies are linear combinations of each other results in the treatment efficacy also being linearly additive. Specifically, biofilms treated with the SP-BE, whose energy is the sum of the energy of the AC-BE and DC-BE, results in a net efficacy that is not significantly different from the linear sum of the efficacy owing to the AC-BE and DC-BE. We also demonstrate that treatment with an AC, DC or SP signal of equivalent energies results in the similar treatment efficacies, at potentials less than 1 V. We further confirm that the relationship between the applied energy to the BE treatment and the total biomass is linear. This linear relationship was verified by measuring total biomass after treatment with electrical signals of varying potentials of both different (AC, DC and SP) and similar signal types (AC signal only). These results when taken together suggest that the magnitude of the electrical energy of the BE is the driving force in determining the efficacy of treatment at such low voltages. This suggests that at potentials below bulk media electrolysis, the mechanism of action of the BE treatment is not different for the various types of signals (AC, DC or SP), as reported previously in literature. Importantly, these findings suggest that this method is aptly suited for deployment in clinical biofilm treatment, as it enables more flexibility and ease of integration of the BE into various, especially in *in vivo* environments.

## References

[bib1] Costerton JW , Stewart PS , Greenberg EP . Bacterial biofilms: a common cause of persistent infections. Science 1999; 284: 1318–1322. 10334980 10.1126/science.284.5418.1318

[bib2] Darouiche RO . Treatment of infections associated with surgical implants. N Engl J Med 2004; 350: 1422–1429. 15070792 10.1056/NEJMra035415

[bib3] Ghannoum M , O'Toole GA . Microbial Biofilms. ASM Press: Washington D.C., USA, 2004.

[bib4] Stoodley P , Sauer K , Davies D , Costerton JW . Biofilms as complex differentiated communities. Annu Rev Microbiol 2002; 56: 187–209. 12142477 10.1146/annurev.micro.56.012302.160705

[bib5] Characklis W . Bioengineering report: Fouling biofilm development—a process analysis. Biotechnol Bioeng 1981; 23: 1923–1960. 10.1002/bit.22227 19090542

[bib6] Fux CA , Stoodley P , Hall-Stoodley L , Costerton JW . Bacterial biofilms: a diagnostic and therapeutic challenge. Expert Rev Anti Infect Ther 2003; 1: 667–683. 15482163 10.1586/14787210.1.4.667

[bib7] Al-Nasiry S , Geusens N , Hanssens M , Luyten C , Pijnenborg R . The use of Alamar Blue assay for quantitative analysis of viability, migration and invasion of choriocarcinoma cells. Hum Reprod 2007; 22: 1304–1309. 17307808 10.1093/humrep/dem011

[bib8] Pozo J , Patel R . The challenge of treating biofilm‐associated bacterial infections. Clin Pharmacol Ther 2007; 82: 204–209. 17538551 10.1038/sj.clpt.6100247

[bib9] Costerton JW , Ellis B , Lam K , Johnson F , Khoury AE . Mechanism of electrical enhancement of efficacy of antibiotics in killing biofilm bacteria. Antimicrob Agents Chemother 1994; 38: 2803–2809. 7695266 10.1128/aac.38.12.2803 PMC188289

[bib10] Efron D , Jarman F , Barker M . Methylphenidate versus dexamphetamine in children with attention deficit hyperactivity disorder: a double-blind, crossover trial. Pediatrics 1997; 100: e6–e. 10.1542/peds.100.6.e6 9382907

[bib11] Hoffman LR , D'Argenio DA , MacCoss MJ , Zhang Z , Jones RA , Miller SI . Aminoglycoside antibiotics induce bacterial biofilm formation. Nature 2005; 436: 1171–1175. 16121184 10.1038/nature03912

[bib12] Cottarel G , Wierzbowski J . Combination drugs, an emerging option for antibacterial therapy. Trends Biotechnol 2007; 25: 547–555. 17997179 10.1016/j.tibtech.2007.09.004

[bib13] Pareilleux A , Sicard N . Lethal effects of electric current on *Escherichia coli*. Appl Microbiol 1970; 19: 421–424. 4909349 10.1128/am.19.3.421-424.1970 PMC376703

[bib14] Blenkinsopp SA , Khoury A , Costerton J . Electrical enhancement of biocide efficacy against *Pseudomonas aeruginosa* biofilms. Appl Environ Microbiol 1992; 58: 3770–3773. 1482196 10.1128/aem.58.11.3770-3773.1992 PMC183173

[bib15] Khoury AE , Lam K , Ellis B , Costerton JW . Prevention and control of bacterial infections associated with medical devices. ASAIO J 1992; 38: M174–M178. 1457842 10.1097/00002480-199207000-00013

[bib16] Wellman N , Fortun SM , McLeod BR . Bacterial biofilms and the bioelectric effect. Antimicrob Agents Chemother 1996; 40: 2012–2014. 8878572 10.1128/aac.40.9.2012 PMC163464

[bib17] Stoodley P , deBeer D , Lappin-Scott HM . Influence of electric fields and pH on biofilm structure as related to the bioelectric effect. Antimicrob Agents Chemother 1997; 41: 1876–1879. 9303377 10.1128/aac.41.9.1876 PMC164028

[bib18] Stewart PS , Wattanakaroon W , Goodrum L , Fortun SM , McLeod BR . Electrolytic generation of oxygen partially explains electrical enhancement of tobramycin efficacy against *Pseudomonas aeruginosa* biofilm. Antimicrob Agents Chemother 1999; 43: 292–296. 9925521 10.1128/aac.43.2.292 PMC89066

[bib19] Caubet R , Pedarros-Caubet F , Chu M , Freye E , de Belem Rodrigues M , Moreau J et al. A radio frequency electric current enhances antibiotic efficacy against bacterial biofilms. Antimicrob Agents Chemother 2004; 48: 4662–4664. 15561841 10.1128/AAC.48.12.4662-4664.2004 PMC529182

[bib20] Shirtliff ME , Bargmeyer A , Camper AK . Assessment of the ability of the bioelectric effect to eliminate mixed-species biofilms. Appl Environ Microbiol 2005; 71: 6379–6382. 16204561 10.1128/AEM.71.10.6379-6382.2005 PMC1265951

[bib21] Del Pozo JL , Rouse MS , Euba G , Kang C-I , Mandrekar JN , Steckelberg JM et al. The electricidal effect is active in an experimental model of *Staphylococcus epidermidis* chronic foreign body osteomyelitis. Antimicrob Agents Chemother 2009; 53: 4064–4068. 19651912 10.1128/AAC.00432-09 PMC2764171

[bib22] Del Pozo JL , Rouse MS , Mandrekar JN , Sampedro MF , Steckelberg JM , Patel R . Effect of electrical current on the activities of antimicrobial agents against *Pseudomonas aeruginosa*, *Staphylococcus aureus*, and *Staphylococcus epidermidis* biofilms. Antimicrob Agents Chemother 2008; 53: 35–40. 18725436 10.1128/AAC.00237-08 PMC2612137

[bib23] Del Pozo JL , Rouse MS , Mandrekar JN , Steckelberg JM , Patel R . The electricidal effect: reduction of *Staphylococcus* and *Pseudomonas* biofilms by prolonged exposure to low-intensity electrical current. Antimicrob Agents Chemother 2009; 53: 41–45. 18955534 10.1128/AAC.00680-08 PMC2612149

[bib24] Giladi M , Porat Y , Blatt A , Shmueli E , Wasserman Y , Kirson ED et al. Microbial growth inhibition by alternating electric fields in mice with *Pseudomonas aeruginosa* lung infection. Antimicrob Agents Chemother 2010; 54: 3212–3218. 20547811 10.1128/AAC.01841-09 PMC2916302

[bib25] Sandvik EL , McLeod BR , Parker AE , Stewart PS . Direct electric current treatment under physiologic saline conditions kills *Staphylococcus epidermidis* biofilms via electrolytic generation of hypochlorous acid. PLoS One 2013; 8: e55118. 23390518 10.1371/journal.pone.0055118 PMC3563656

[bib26] Del Pozo JL , Rouse MS , Patel R . Bioelectric effect and bacterial biofilms. A systematic review. Int J Artif Organs 2008; 31: 786–795. 18924090 10.1177/039139880803100906 PMC3910516

[bib27] Van Der Borden AJ , Van Der Werf H , Van Der Mei HC , Busscher HJ . Electric current-induced detachment of *Staphylococcus epidermidis* biofilms from surgical stainless steel. Appl Environ Microbiol 2004; 70: 6871–6874. 15528555 10.1128/AEM.70.11.6871-6874.2004 PMC525258

[bib28] Wang L , Li J , March JC , Valdes JJ , Bentley WE . luxS-dependent gene regulation in Escherichia coli K-12 revealed by genomic expression profiling. J Bacteriol 2005; 187: 8350–8360. 16321939 10.1128/JB.187.24.8350-8360.2005 PMC1316998

[bib29] Roy V , Meyer MT , Smith JAI , Gamby S , Sintim HO , Ghodssi R et al. AI-2 analogs and antibiotics: a synergistic approach to reduce bacterial biofilms. Appl Microbiol Biotechnol 2013; 97: 2627–2638. 23053069 10.1007/s00253-012-4404-6

[bib30] Jun W , Kim MS , Cho B-K , Millner PD , Chao K , Chan DE . Microbial biofilm detection on food contact surfaces by macro-scale fluorescence imaging. J Food Eng 2010; 99: 314–322.

[bib31] Merritt JH , Kadouri DE , O'Toole GA . Growing and analyzing static biofilms. In: Current Protocols in Microbiology. John Wiley & Sons, Inc.: Hoboken, NJ, USA, 2005. 10.1002/9780471729259.mc01b01s00 PMC4568995 18770545

[bib32] Donlan RM . Biofilms and device-associated infections. Emerg Infect Dis 2001; 7: 277–281. 11294723 10.3201/eid0702.010226 PMC2631701

[bib33] Bockris JOM , Reddy AK . Modern Electrochemistry. Kluwer Acadamic/Plenum Publishers: New York, NY, USA, 2000.

[bib34] Neagu C , Jansen H , Gardeniers H , Elwenspoek M . The electrolysis of water: an actuation principle for MEMS with a big opportunity. Mechatronics 2000; 10: 571–581.

[bib35] Lathi BP . Modern Digital and Analog Communication Systems. Oxford University Press, Inc.: New York, NY, USA, 1990.

[bib36] Mah T-FC , O'Toole GA . Mechanisms of biofilm resistance to antimicrobial agents. Trends Microbiol 2001; 9: 34–39. 11166241 10.1016/s0966-842x(00)01913-2

[bib37] Anderson GG , O'toole GA . Innate and induced resistance mechanisms of bacterial biofilms. In: Bacterial Biofilms. Springer: Berlin, Germany, 2008, pp 85–105. 10.1007/978-3-540-75418-3_5 18453273

[bib38] Anwar H , Dasgupta MK , Costerton JW . Testing the susceptibility of bacteria in biofilms to antibacterial agents. Antimicrob Agents Chemother 1990; 34: 2043. 2073094 10.1128/aac.34.11.2043 PMC171995

[bib39] Gyurova AY , Zhivkov AM . Influence of the medium electrolyte concentration on the electric polarizability of bacteria *Escherichia coli* in presence of ethanol. Colloids Surf B Biointerfaces 2009, 11/1/ 74: 23–27. 19631513 10.1016/j.colsurfb.2009.06.017

[bib40] Zwietering M , De Koos J , Hasenack B , De Witt J , Van't Riet K . Modeling of bacterial growth as a function of temperature. Appl Environ Microbiol 1991; 57: 1094–1101. 2059034 10.1128/aem.57.4.1094-1101.1991 PMC182851

[bib41] Jass J , Costerton J , Lappin-Scott H . The effect of electrical currents and tobramycin on *Pseudomonas aeruginosa* biofilms. J Ind Microbiol 1995; 15: 234–242. 8519482 10.1007/BF01569830

[bib42] Jass J , Lappin-Scott HM . The efficacy of antibiotics enhanced by electrical currents against *Pseudomonas aeruginosa* biofilms. J Antimicrob Chemother 1996; 38: 987–1000. 9023646 10.1093/jac/38.6.987

[bib43] Freebairn D , Linton D , Harkin-Jones E , Jones DS , Gilmore BF , Gorman SP . Electrical methods of controlling bacterial adhesion and biofilm on device surfaces. Expert Rev Med Devices 2013; 10: 85–103. 23278226 10.1586/erd.12.70

