# Supplementary material for: Effect of electrical energy on the efficacy of biofilm treatment using the bioelectric effect
Source: NPJ Biofilms Microbiomes. 2015 Sep 23;1:15016–. doi: 10.1038/npjbiofilms.2015.16 (PMC5515217; doi:10.1038/npjbiofilms.2015.16)
Supplement: Supplementary Figure S1 [file npjbiofilms201516-s1.doc]

**Supplemental Material:**


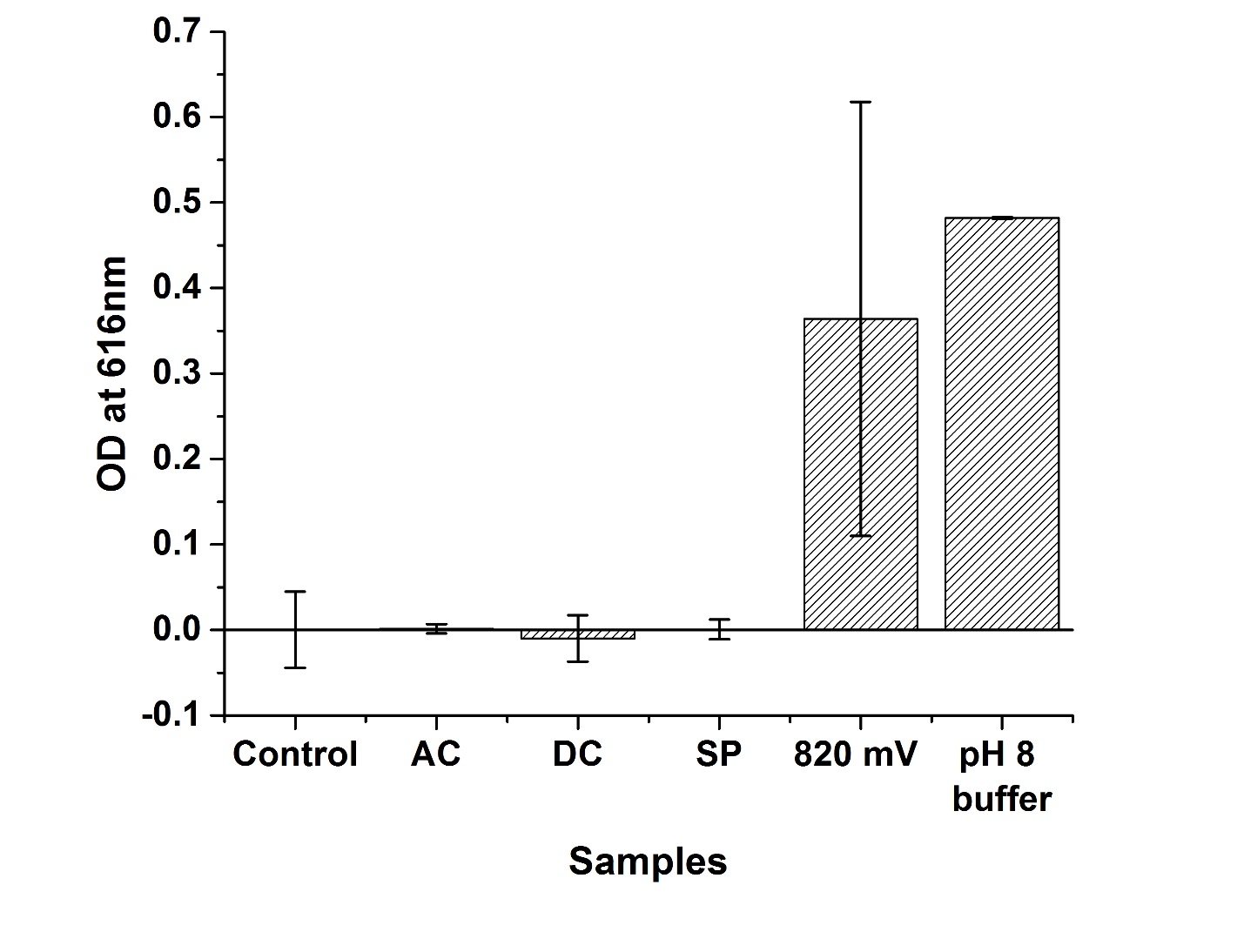


Figure S1: Results of bulk electrolysis quantification using a pH indicator (N=3). 1 mL of fresh unbuffered LB growth media was placed in the cuvette and electrical potentials (no antibiotics) were applied for 24 hours. The potentials applied are: AC: 0.5 V at 10 MHz; DC: 0.5 V; SP: 0.5 V DC + 0.5 V at 10MHz; and the electrolysis threshold voltage of 0.82 V DC. No electrical potentials were applied to the controls (pure LB media or pH 8 phosphate buffer). Since electrolysis induces the production of hydrogen gas, the solution is expected to become more basic^18, 26, 34^. This increase in pH is used to indirectly quantify the bulk electrolysis of the media due to application of electrical potentials. Following 24 hours of electrical potential application, two drops of the pH indicator were added to the solution and the OD spectrum was measured by the spectrophotometer. Since the peak value of the pH 8 buffer solution using the pH indicator was observed at 616 nm wavelength, OD_616_ was selected to quantify the electrolysis effect. As observed, only the samples to which 0.82 V was applied and the pH8 buffer positive control samples showed significantly higher OD compared to other treatments. We thus conclude that application of low energy electrical fields does not induce significant bulk electrolysis.
